# Modeling of dielectric resonator antenna array for retina photoreceptors^☆^

**DOI:** 10.1016/j.heliyon.2023.e13794

**Published:** 2023-02-21

**Authors:** Mahdi NoroozOliaei, Hamid Riazi Esfahani, Mohammad Sadegh Abrishamian

**Affiliations:** a K. N. Toosi University of Technology, Electrical & Computer Engineering Department, Iran; b Tehran University of Medical Sciences (TUMS), Farabi Eye Hospital, Iran

**Keywords:** Cone photoreceptor, Dielectric Resonator Antenna (DRA), Electrochemical signals, Array antenna, mfERG, Outer segment

## Abstract

The retina encompasses several cone and rod photoreceptors at fovea region i.e., 90 million cells of rod photoreceptors and 4.5million cells of cone photoreceptors. The overall photoreceptors determine the vision of every human. An electromagnetic dielectric resonator antenna has been presented for retina photoreceptors in order to model them at fovea and its peripheral retina with the respected angular spectrum. Three coloring primary system of human eye (R, G, B) can be realized based on the model. Three miscellaneous models i.e., simple, graphene coated, and interdigital models have been presented in this paper. The nonlinear property of interdigital structures is one of the best advantages to use for creating the capacitor. The capacitance property helps improving the upper band of visible spectrum. The absorption of light for graphene as an energy harvesting material and its conversion into electrochemical signals is making it one of the best models. The mentioned three electromagnetic models of human photoreceptors have been expressed as a receiver antenna. The proposed electromagnetic models based on dielectric resonator antenna (DRA) are being analyzed for cones and rods photoreceptors of retina in the human eye by Finite Integral Method (FIM) utilized by CST MWS. The results show that the models are so fine for vision spectrum due to its localized near field enhancement property. The results indicate fine parameters of $S_{11}$ (return loss below -10 dB) with invaluable resonants in a wide range of frequencies from 405 THz to 790 THz (vision spectrum), appropriate $S_{21}$ (insertion loss 3-dB bandwidth), very good field distribution of electric and magnetic fields for flowing the power and electrochemical signals. Finally, mfERG clinical and experimental results validate the numeric results by the normalized output to input ratio of these models and it points out that these models can stimulate the electrochemical signals in photoreceptor cells for the best suiting of realizing the new retinal implants.

## 1 Introduction

Stimulation of nerves for eyes to recover the lost vision is necessary for patients. The electromagnetic modeling of retina photoreceptors is the best way to realize this vital procedure. Photoreceptors in a normal eye are being located at the outer layers of retina containing photopigments which they are responsible for phototransduction to generate the electrochemical neuronal signals in the presence of light stimuli. These signals can be passed and processed by some neurons at the middle layers of the retina before reaching the retinal ganglion cells (RGC) within the inner layers of retina and the central nervous system (CNS) by optic nerve. Vision restoration of blind people can be achieved by creating devices such as retinal prostheses which they can receive the light and process it. After processing the light, the information can be transmitted in the form of electrical impulses to the remaining inner retinal layers for the visual function. One of the big electromagnetic challenges is analysis and modeling of retina and its optic nerve in order to replace a prosthesis as a bionic eye. Many good endeavors for realizing commercial prosthesis (epi-retinal and sub-retinal implants) are being done so far [1], [2], [3], [4], [5], [6], [7]. In [8], a graphene based antenna model has been introduced for the electromagnetic model of retina photoreceptors. The typical human trichromatic color visual system combines the response of three types of cone cells in retina as shown in Fig. 1 of [8]. In this paper, the proposed electromagnetic models on array antenna of different models of dielectric resonator antenna (DRA) are being simulated for cones and rods in retina photoreceptors of human eye by Finite Integral Method (FIM) utilizing CST MWS.

**Figure 1:**
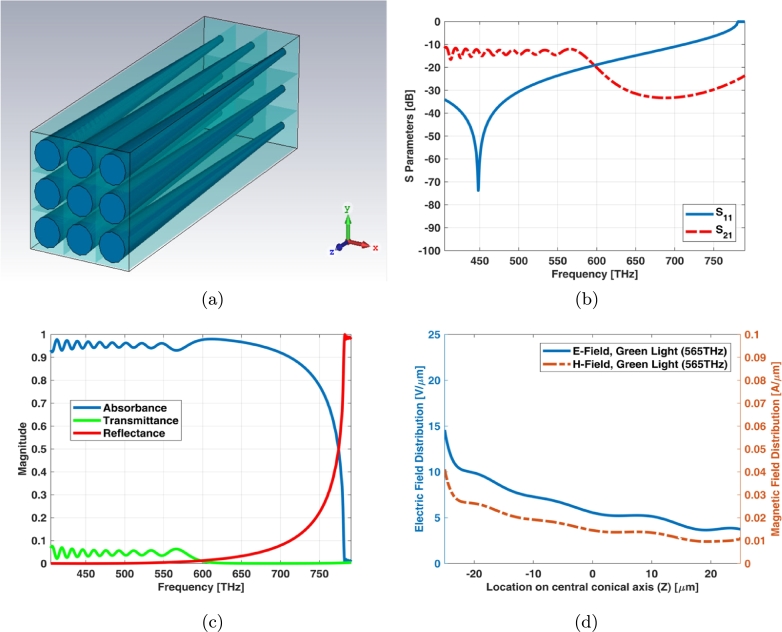
3 × 3 array of simple cone photoreceptor (a) schematic, (b) S parameters, (c) reflectance, transmittance and absorbance index, and (d) electric and magnetic fields in one sample frequency - green light (565 THz).

Two modes of array antenna have been investigated in this paper. Finite array with 9 (3 × 3) elements and infinite array (unit cell boundary conditions). There are three different models: simple, graphene coated and interdigital one. The dielectric constant of photoreceptor (cone or rod shapes) and its media (with a cross section of 5 × 5 ${\mu \text{m}}^{2}$) are $\epsilon_{r_{Ph}}$ =2.1 and $\epsilon_{r_{M}}$ =1.85, respectively. The description of human cone cells dimension has been presented in [8].

The dimensions of human rod cell are being brought into [9] as well.

The distributions of photoreceptors in the retina are defined as the following:

1- Cone photoreceptors at the central region (from $0^{\circ }$ to $5^{\circ }$) of retina (fovea) with a high density which it is defined by infinite array (unit cell).

2- Cone photoreceptors at the near central region (from $5^{\circ }$ to $15^{\circ }$) of retina with a moderated density which it can be shown by finite 3 × 3 array.

3- Rod photoreceptors (from $20^{\circ }$-$27^{\circ }$) of retina with a high density which it is defined by infinite array (unit cell).

4- Rod photoreceptors (more than $27^{\circ }$) of retina with a moderated density which it can be replaced by finite 3 × 3 array.

Every simulated cone photoreceptor array has the equal length of 50 μm and the diameter varies from 4 μm (first point) to 1 μm (end point). Every simulated rod photoreceptor array has the equal length of 100 μm and the diameter is 2 μm. The results of the output to input ratio obtained by numerical method of the mentioned arrangement for photoreceptors array have very good conformance with mfERG ones of a normal subject with a valid interpretation of the signal to noise ratio of the experiments.

The following sections illustrate the response and electromagnetic fields at one frequency sample of each three models.

## 2 Array of simple model (equal cone photoreceptor)

3 × 3 array of simple cone photoreceptor model has been illustrated in Fig. 1(a). The array assumed to be equal in size (both length and width). S parameters (return loss and insertion loss) and reflection, absorption, and transmission indexes of this array have been shown in Figs. 1(b) and 1(c). The main resonant frequency of finite cone photoreceptor array is around 450 THz (Red Region). It is noteworthy that these types of structures (Dielectric Resonant Antenna) have several resonant frequencies at visible spectrum. It has no significant transmission coefficient. The absorption is high up to high frequencies around 780 THz and the reflection increases after frequencies around 650 THz. It will be reached to its maximum at frequencies around 780 THz. Fig. 1(d) shows electric and magnetic fields of 3 × 3 array for simple cone photoreceptor in its maximum possibility of local magnitude at one of frequency samples i.e., 565 THz.

## 3 Array of simple model (equal rod photoreceptor)

**Figure 2:**
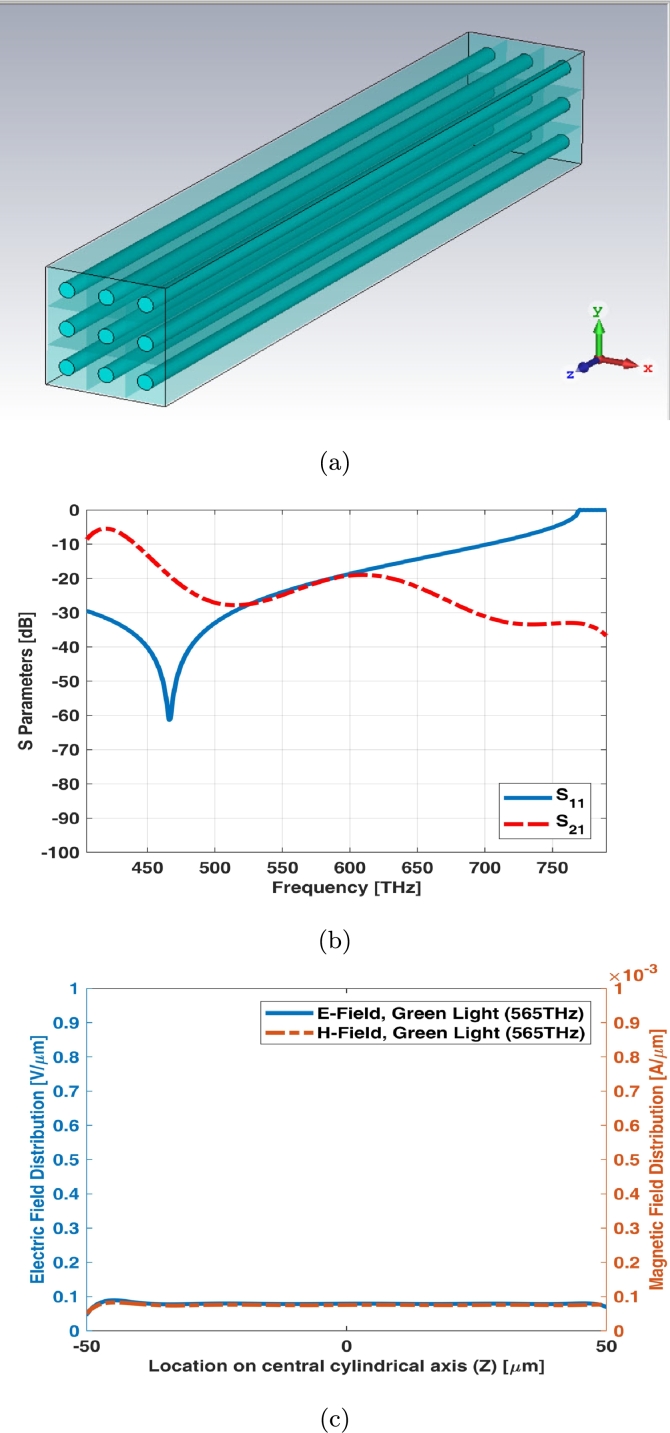
3 × 3 array of simple rod photoreceptor (a) schematic, (b) S parameters, and (c) electric and magnetic fields in one sample frequency - green light (565 THz).

3 × 3 array of simple rod photoreceptor model has been illustrated in Fig. 2(a). The array assumed to be equal in size (both length and width). S parameters (return loss and insertion loss) of this array have been shown in Fig. 2(b). There is no significant transmission for rod photoreceptors as shown in Fig. 2(b). It is also fine for these types of photoreceptors as rod cell receives lower luminance of signals in scotopic vision. In other words, it requires less light to function than cone. Fig. 2(c) shows electric and magnetic fields of 3 × 3 array for simple rod photoreceptor at one of frequency samples i.e., 565 THz.

## 4 Unit cell of simple model (cone photoreceptor)

**Figure 3:**
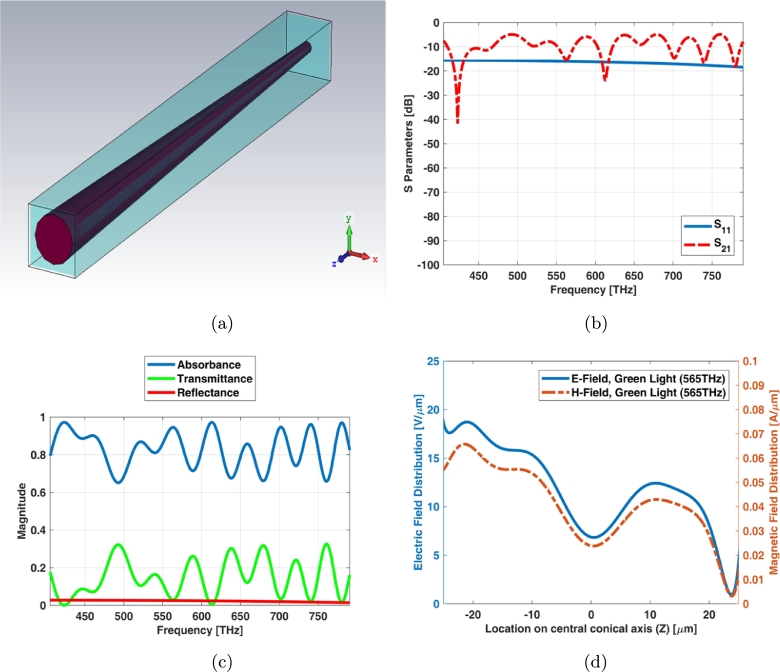
Unit cell of simple cone photoreceptor (a) schematic, (b) S parameters, (c) reflectance, transmittance and absorbance index, and (d) electric and magnetic fields in one sample frequency - green light (565 THz).

The unit cell boundary condition provides criterion which it equals to infinite array. Simple cone photoreceptor infinite array (unit cell) has the equal length and diameters as shown in Fig. 3(a). The return loss ($S_{11}$) of unit cell is below -15 dB while the insertion loss ($S_{21}$) has a ripple shape at visible spectrum (Fig. 3(b)). It is also obvious from transmittance and absorbance indexes in Fig. 3(c). Fig. 3(d) shows electric and magnetic fields of unit cell (infinite array) for simple cone photoreceptor at one of frequency samples i.e., 565 THz.

## 5 Unit cell of simple model (rod photoreceptor)

**Figure 4:**
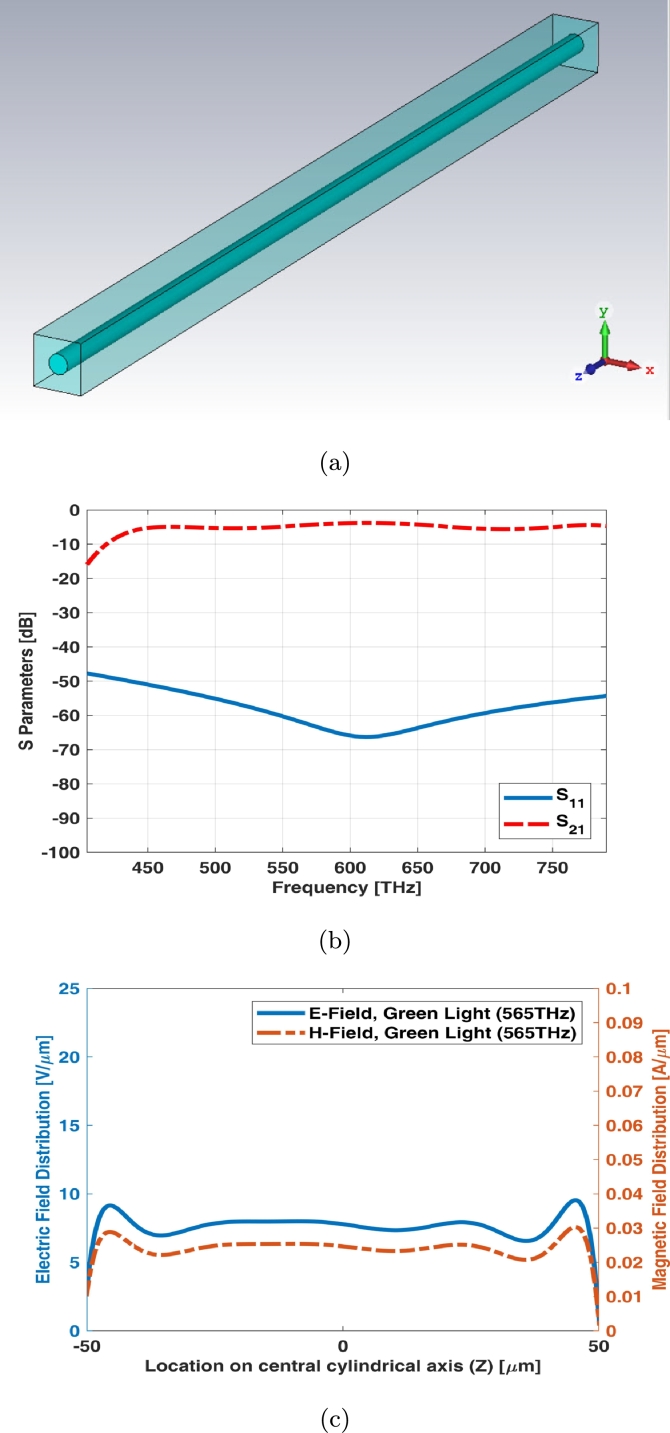
Unit cell of simple rod photoreceptor (a) schematic, (b) S parameters, and (c) electric and magnetic fields in one sample frequency - green light (565 THz).

Simple rod photoreceptor infinite array (unit cell) has the equal length and the diameter as shown in Fig. 4(a). The return loss ($S_{11}$) of unit cell shows main resonant at middle frequency of visible spectrum while the insertion loss ($S_{21}$) has a slight ripple at visible spectrum (Fig. 4(b)). Fig. 4(c) shows electric and magnetic fields of unit cell (infinite array) for simple rod photoreceptor at one of frequency samples i.e., 565 THz.

## 6 Array of graphene coated model (equal cone photoreceptor)

**Figure 5:**
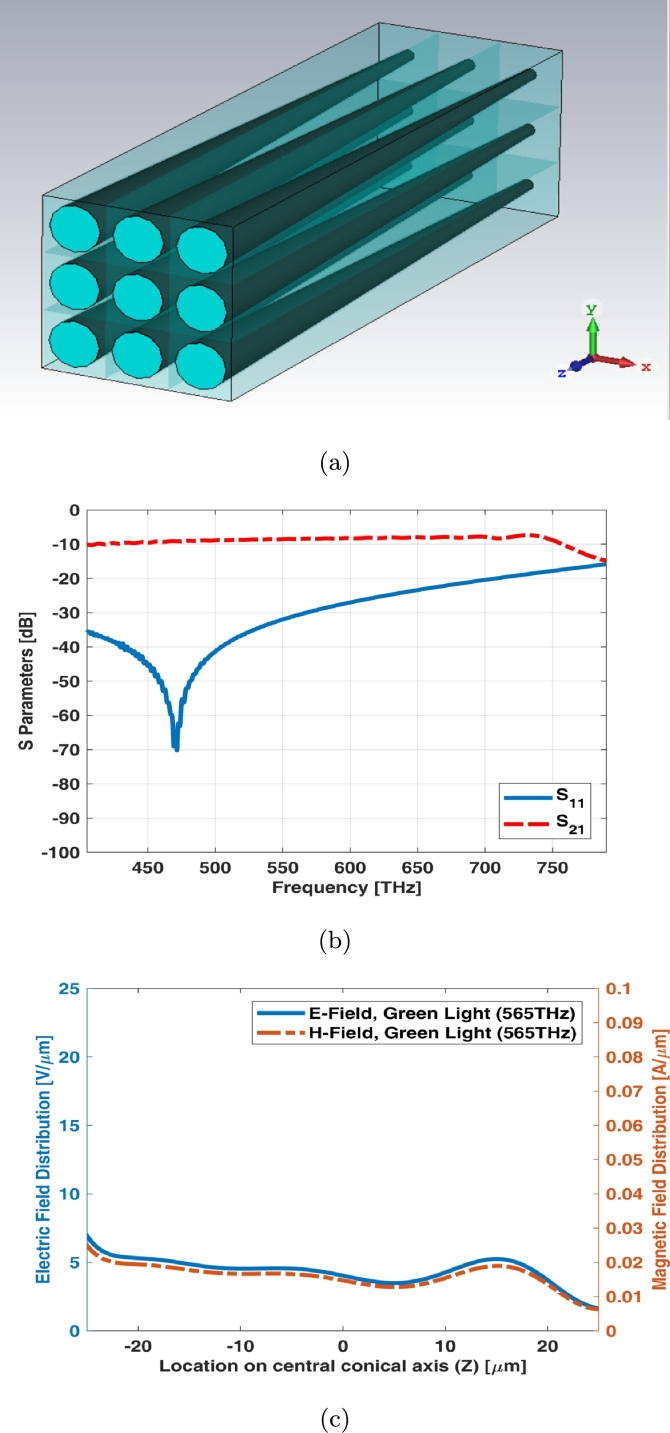
3 × 3 array of graphene coated cone photoreceptor (a) schematic, (b) S parameters, and (c) electric and magnetic fields in one sample frequency - green light (565 THz).

3 × 3 array of graphene coated cone photoreceptor model has been illustrated in Fig. 5(a). The array assumed to be equal in size (both length and width). A thin graphene layer is being coated around the photoreceptor. S parameters (return loss and insertion loss) have been shown in Fig. 5(b). The return loss ($S_{11}$) of 3 × 3 array of graphene coated cone photoreceptor is below -10 dB at visible spectrum and the insertion loss ($S_{21}$) has a slight ripple shape near -10 dB at this spectrum. Fig. 5(c) shows electric and magnetic fields of 3 × 3 array for graphene coated cone photoreceptor at one of frequency samples i.e., 565 THz.

## 7 Array of graphene coated model (equal rod photoreceptor)

**Figure 6:**
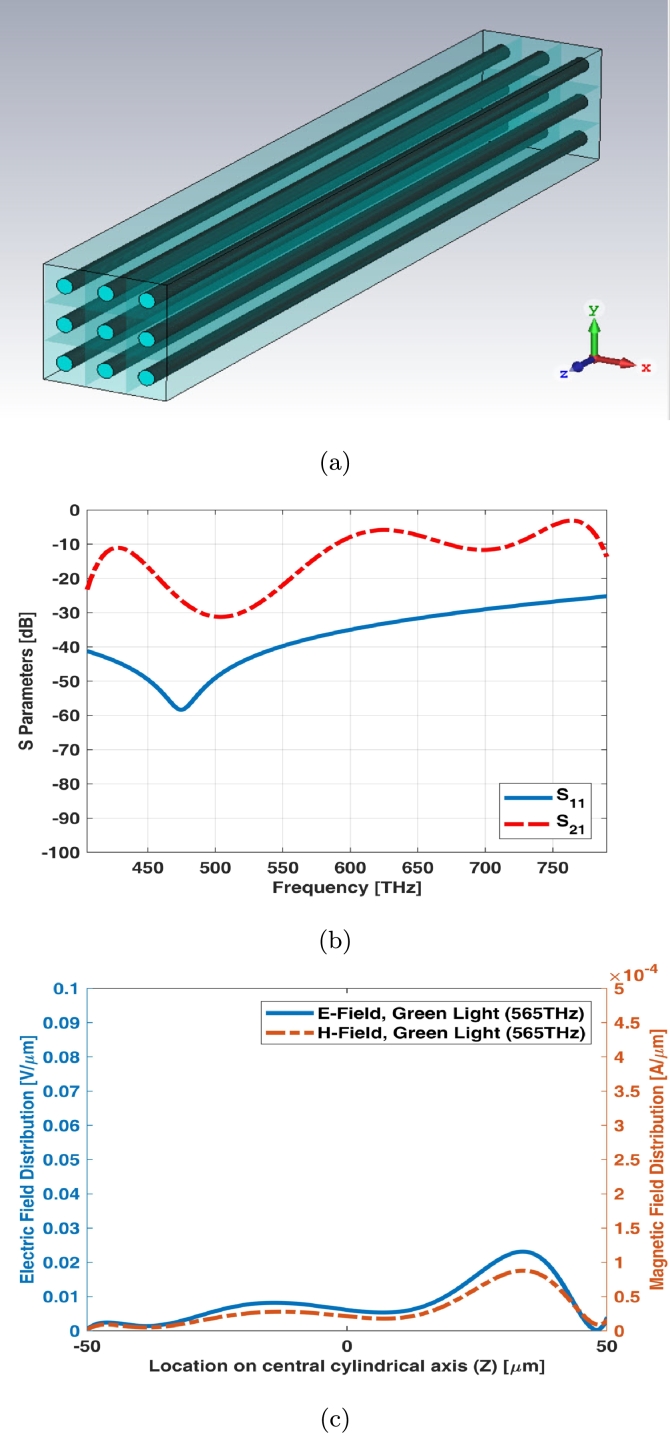
3 × 3 array of graphene coated rod photoreceptor (a) schematic, (b) S parameters, and (c) electric and magnetic fields in one sample frequency - green light (565 THz).

3 × 3 array of graphene coated rod photoreceptor model has been illustrated in Fig. 6(a). The array assumed to be equal in size (both length and width). A thin graphene layer is being coated around the photoreceptor. S parameters (return loss and insertion loss) have been shown in Fig. 6(b). The return loss ($S_{11}$) of 3 × 3 array of graphene coated rod photoreceptor is below -10 dB at visible spectrum and the insertion loss ($S_{21}$) has a ripple shape between near -10 dB and -20 dB at this spectrum. Fig. 6(c) shows electric and magnetic fields of 3 × 3 array for graphene coated rod photoreceptor at one of frequency samples i.e., 565 THz.

## 8 Unit cell of graphene coated model (cone photoreceptor)

**Figure 7:**
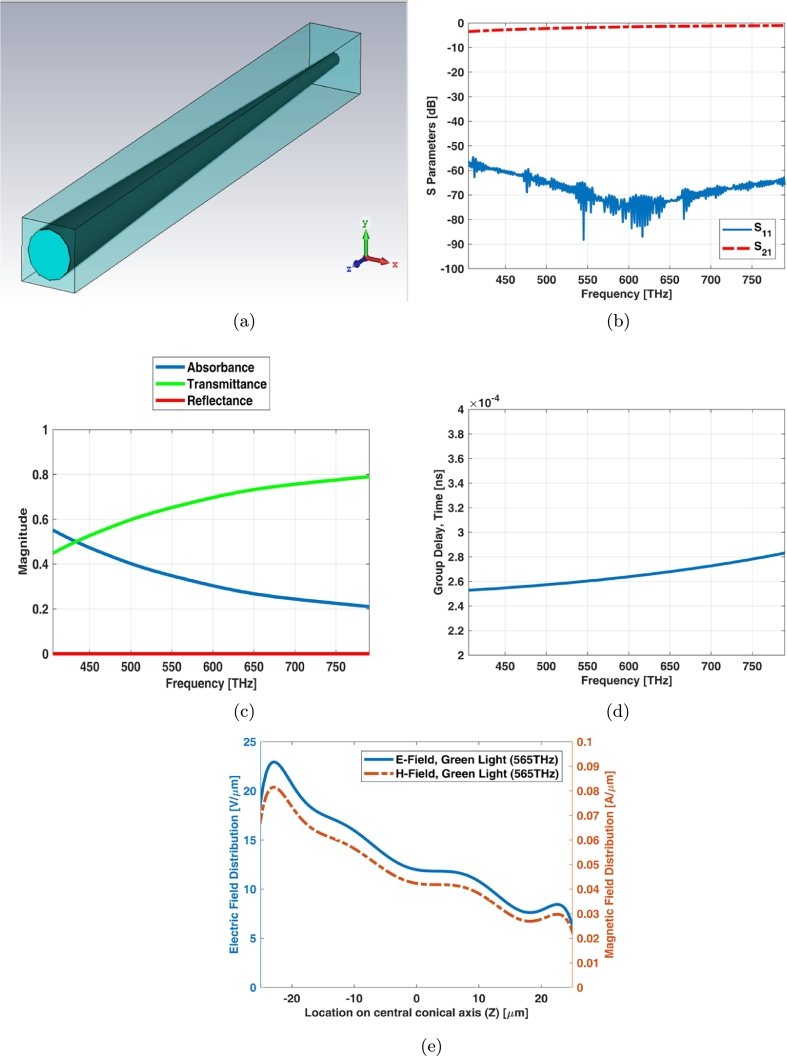
Unit cell of graphene coated cone photoreceptor (a) schematic (b) S parameters, (c) reflectance, transmittance and absorbance index, (d) group delay of transmittance way, and (e) electric and magnetic fields in one sample frequency - green light (565 THz).

Graphene coated cone photoreceptor infinite array (unit cell) has the equal length and the diameter as shown in Fig. 7(a). A thin graphene layer is being coated around the photoreceptor. The main resonant frequencies of unit cell are in the green light spectrum as illustrated in Fig. 7(b)). The transmittance, reflectance and absorbance indexes indicate that this is a proper model for considering as array (Fig. 7(c)). Fig. 7(d) illustrates the group delay of transmitting from port 1 and port 2. Fig. 7(e) shows electric and magnetic fields of unit cell (infinite array) for graphene coated cone photoreceptor at one of frequency samples i.e., 565 THz.

## 9 Unit cell of graphene coated model (rod photoreceptor)

**Figure 8:**
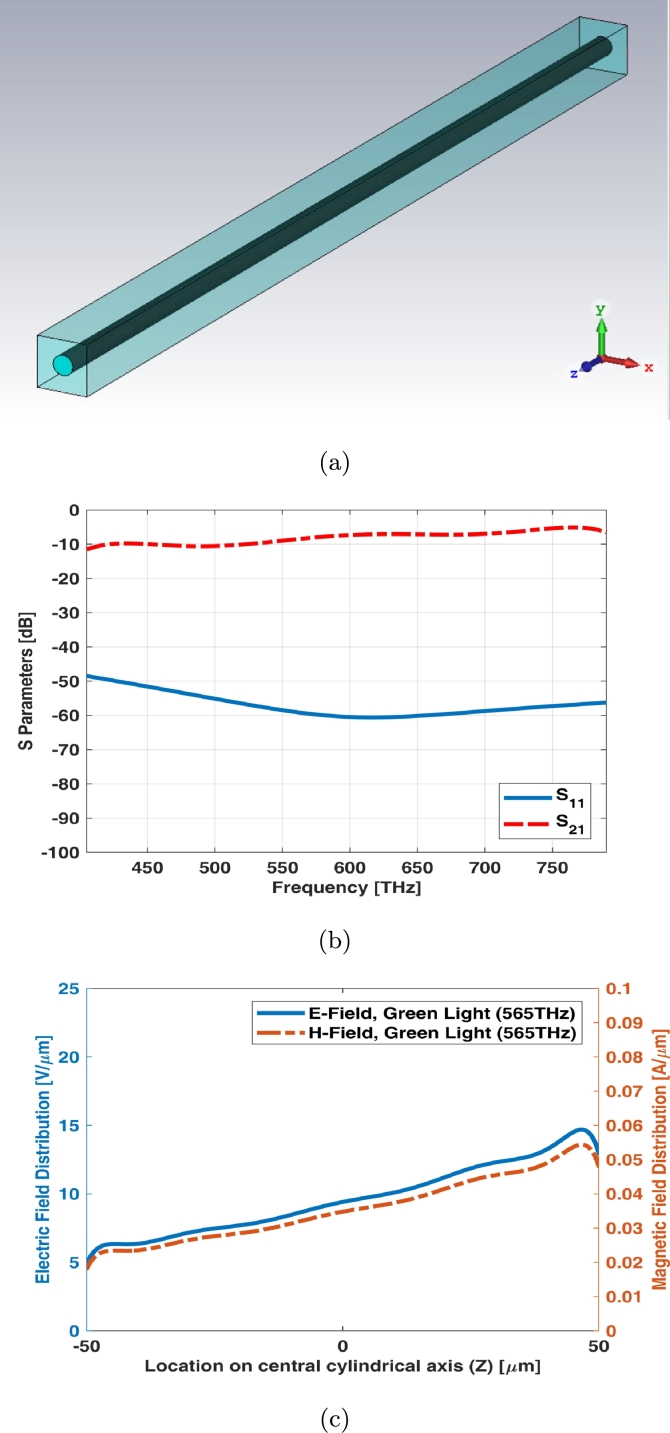
Unit cell of graphene coated rod photoreceptor (a) schematic, (b) S parameters, and (c) electric and magnetic fields in one sample frequency - green light (565 THz).

Graphene coated rod photoreceptor infinite array (unit cell) has the equal length and the diameter as shown in Fig. 8(a). The return loss ($S_{11}$) of unit cell shows that it is below -50 dB while the insertion loss ($S_{21}$) has a slight ripple around -10 dB at visible spectrum (Fig. 8(b)). Fig. 8(c) shows electric and magnetic fields of unit cell (infinite array) for graphene coated rod photoreceptor at one of frequency samples i.e., 565 THz.

## 10 Array of interdigital model (equal cone photoreceptor)

**Figure 9:**
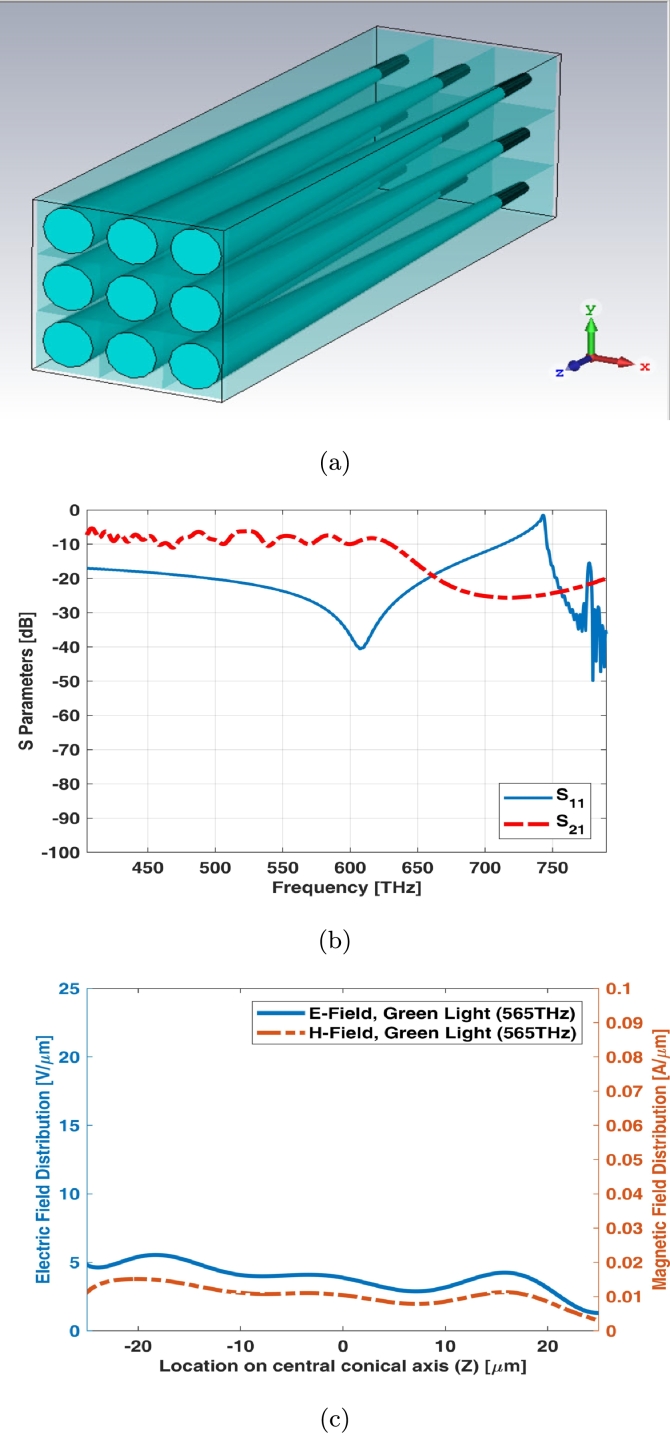
3 × 3 array of interdigital cone photoreceptor (a) schematic, (b) S parameters, and (c) electric and magnetic fields in one sample frequency - green light (565 THz).

3 × 3 array of interdigital cone photoreceptor model has been illustrated in Fig. 9(a). The array assumed to be equal in size (both length and width). S parameters (return loss and insertion loss) have been shown in Fig. 9(b). The main resonant frequencies are in green and blue lights spectrum. The insertion loss ($S_{21}$) has a ripple shape near -10 dB upto 650 THz and then it degrades at higher frequencies of visible spectrum. Fig. 9(c) shows electric and magnetic fields of 3 × 3 array for interdigital cone photoreceptor at one of frequency samples i.e., 565 THz.

## 11 Array of interdigital model (equal rod photoreceptor)

**Figure 10:**
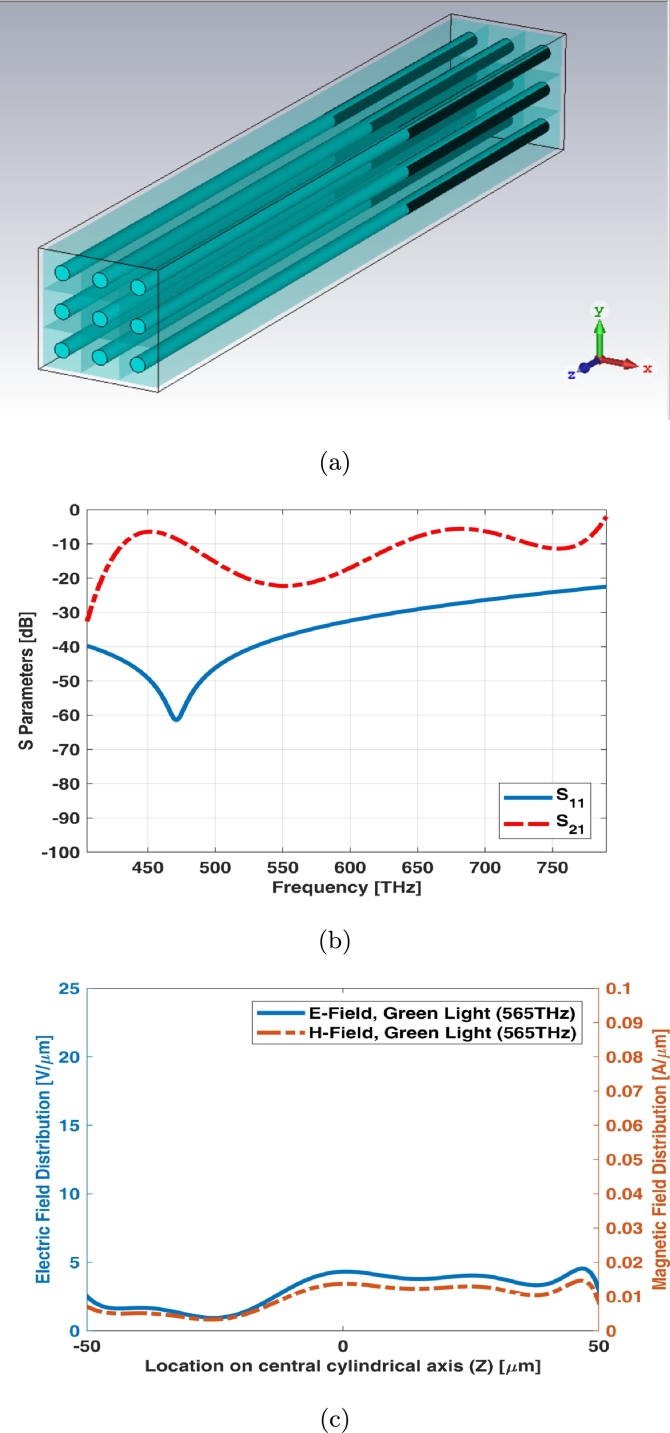
3 × 3 array of interdigital rod photoreceptor (a) schematic, (b) S parameters, and (c) electric and magnetic fields in one sample frequency - green light (565 THz).

3 × 3 array of interdigital rod photoreceptor model has been illustrated in Fig. 10(a). The array assumed to be equal in size (both length and width). S parameters (return loss and insertion loss) have been shown in Fig. 10(b). The return loss ($S_{11}$) is below -20 dB and insertion loss ($S_{21}$) of 3 × 3 array of interdigital rod photoreceptor has a ripple shape around -10 dB to -20 dB at visible spectrum. Fig. 10(c) shows electric and magnetic fields of 3 × 3 array for interdigital rod photoreceptor at one of frequency samples i.e., 565 THz.

## 12 Unit cell of interdigital model (cone photoreceptor)

**Figure 11:**
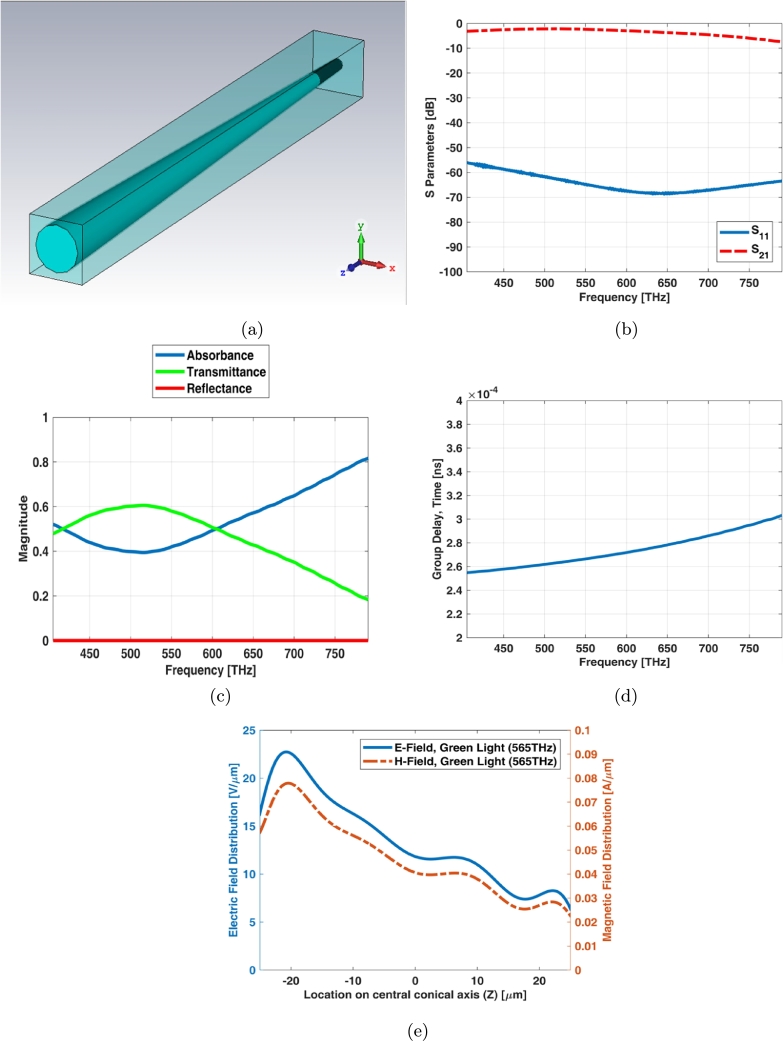
Unit cell of interdigital cone photoreceptor (a) schematic (b) S parameters, (c) reflectance, transmittance and absorbance index, (d) group delay of transmittance way, and (e) electric and magnetic fields in one sample frequency - green light (565 THz).

Interdigital cone photoreceptor infinite array (unit cell) has the equal length and the diameter as shown in Fig. 11(a). The main resonant frequencies of unit cell are in the green light spectrum as illustrated in Fig. 11(b)). The transmittance, reflectance and absorbance indexes indicate that this is a proper model for considering as array (Fig. 11(c)) like the graphene coated model. Fig. 11(d) illustrates the group delay of transmitting from port 1 and port 2 which it shows a low magnitude around the 0.3 ps for determined phase. It can be conformed by N1 implicit time of mfERG technique. Fig. 11(e) shows electric and magnetic fields of unit cell (infinite array) for interdigital cone photoreceptor at one of frequency samples i.e., 565 THz.

## 13 Unit cell of interdigital model (rod photoreceptor)

**Figure 12:**
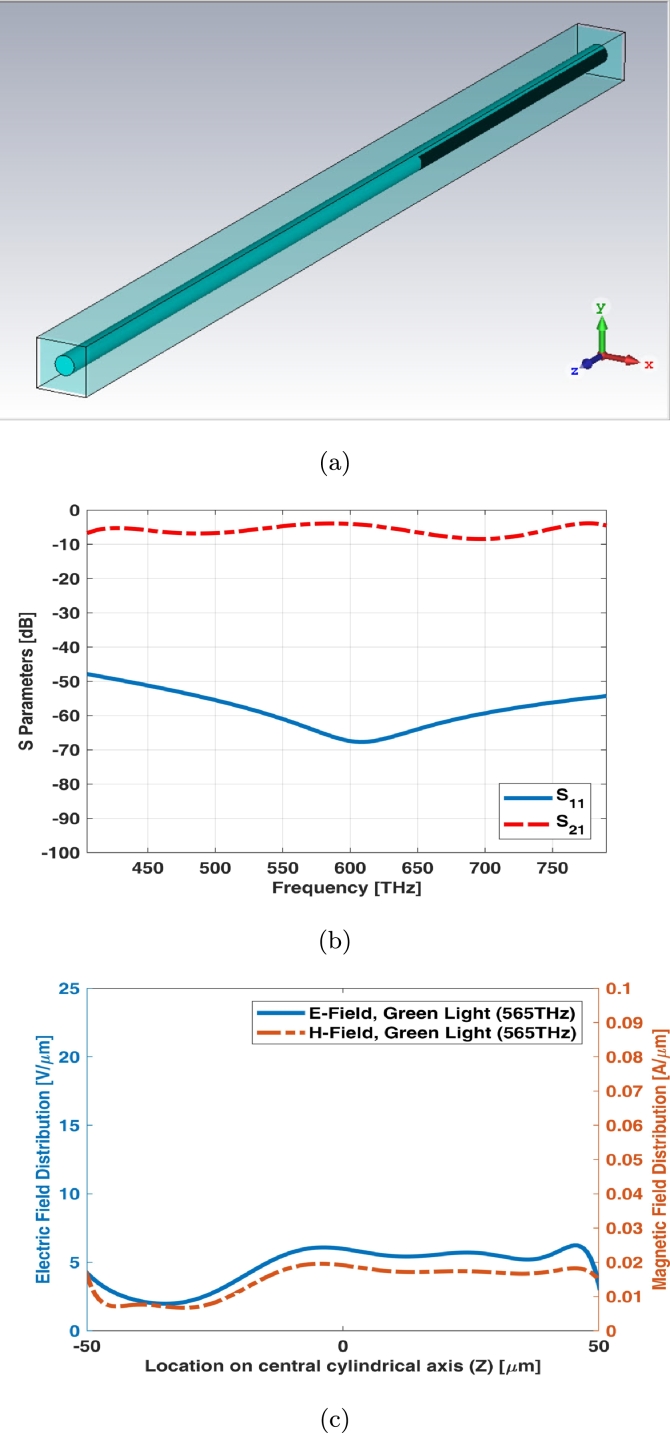
Unit cell of interdigital rod photoreceptor (a) schematic, (b) S parameters, and (c) electric and magnetic fields in one sample frequency - green light (565 THz).

Interdigital rod photoreceptor infinite array (unit cell) has the equal length and the diameter as shown in Fig. 12(a). The return loss ($S_{11}$) of unit cell shows that it has the main resonant in green light frequencies while the insertion loss ($S_{21}$) has a slight ripple around -10 dB at visible spectrum (Fig. 12(b)). Fig. 12(c) shows electric and magnetic fields of unit cell (infinite array) for interdigital rod photoreceptor at one of frequency samples i.e., 565 THz.

## 14 mfERG experimental and clinical results

**Figure 13:**
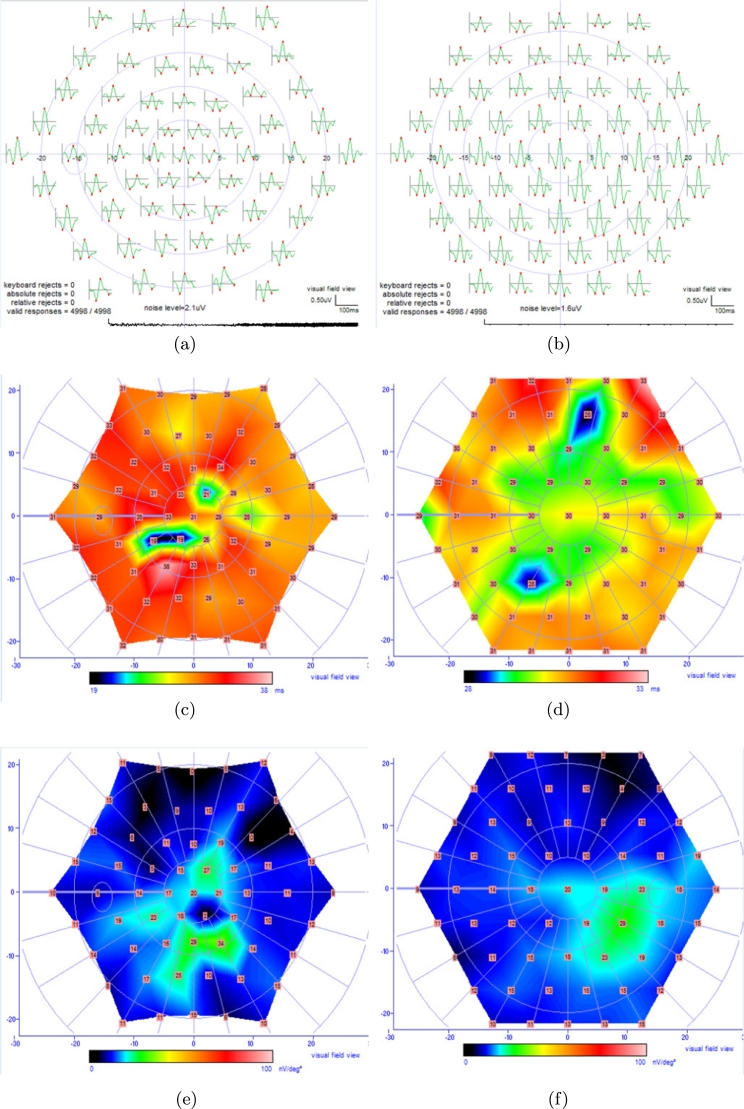
Left eye of a normal subject (a) field trace, (c) N1 implicit time, (e) N1 amplitude, right eye of a normal subject (b) field trace, (d) N1 implicit time, (f) N1 amplitude.

The multifocal ERG responses for a normal subject with a valid interpretation of signal to noise ratio are being shown in Fig. 13 [10], [11], [12], [13], [14], [15]. The field trace, N1 implicit time, and N1 amplitude of left and right eyes are being illustrated in Figs. 13 (a) to (f) correspondingly. The N1 waves are related to the signals of photoreceptors.

## 15 Analysis

**Figure 14:**
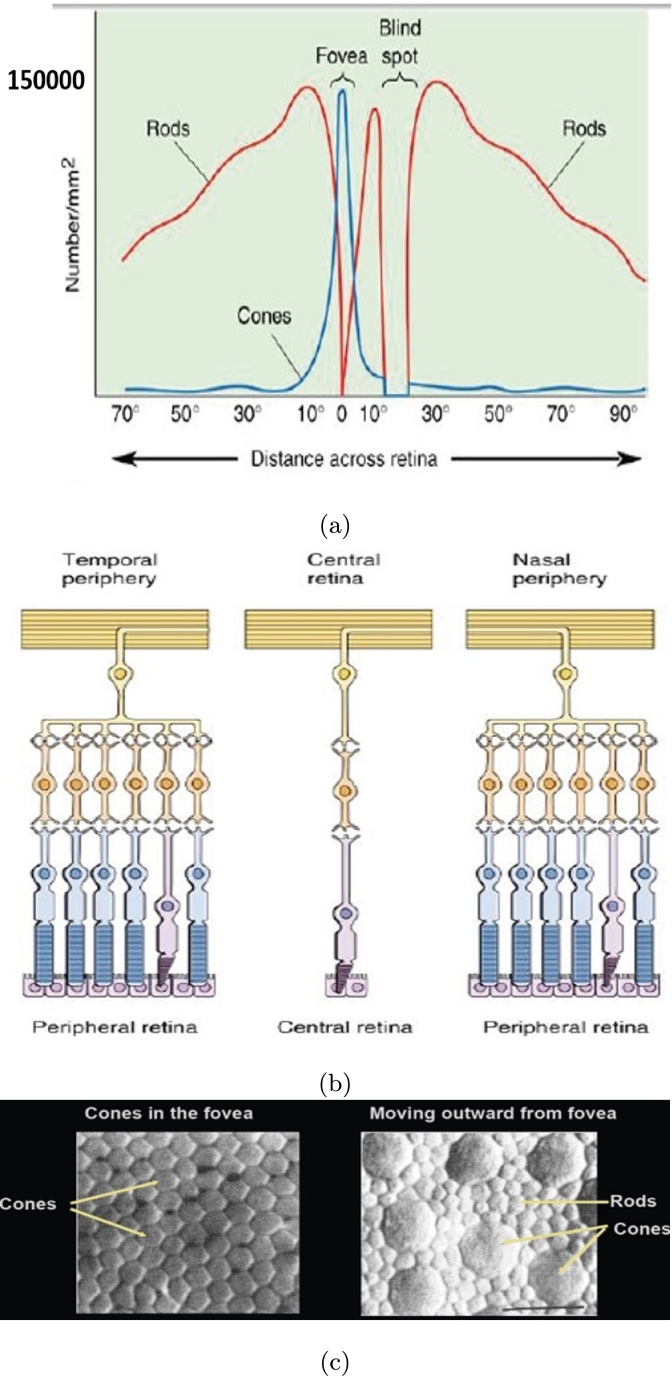
Retina photoreceptors (a) number and distribution of cones and rods in mm^2^ with respect to the distance within retina, (b) illustration of cones and rods connection to the nerves of retina, and (c) illustration of cones and rods (line scale is 10 μm) [16], [17].

**Figure 15:**
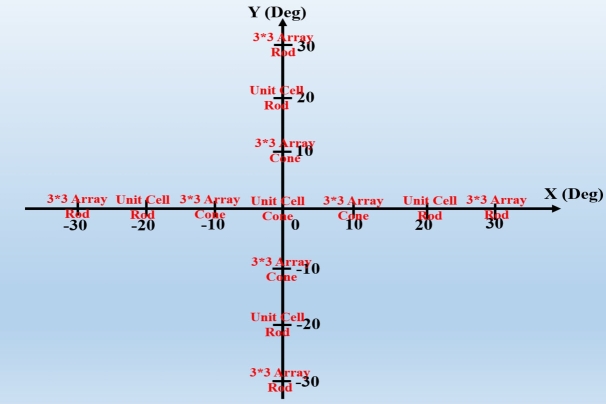
Modeling of retina photoreceptors (cones and rods) in terms of arrays.

**Figure 16:**
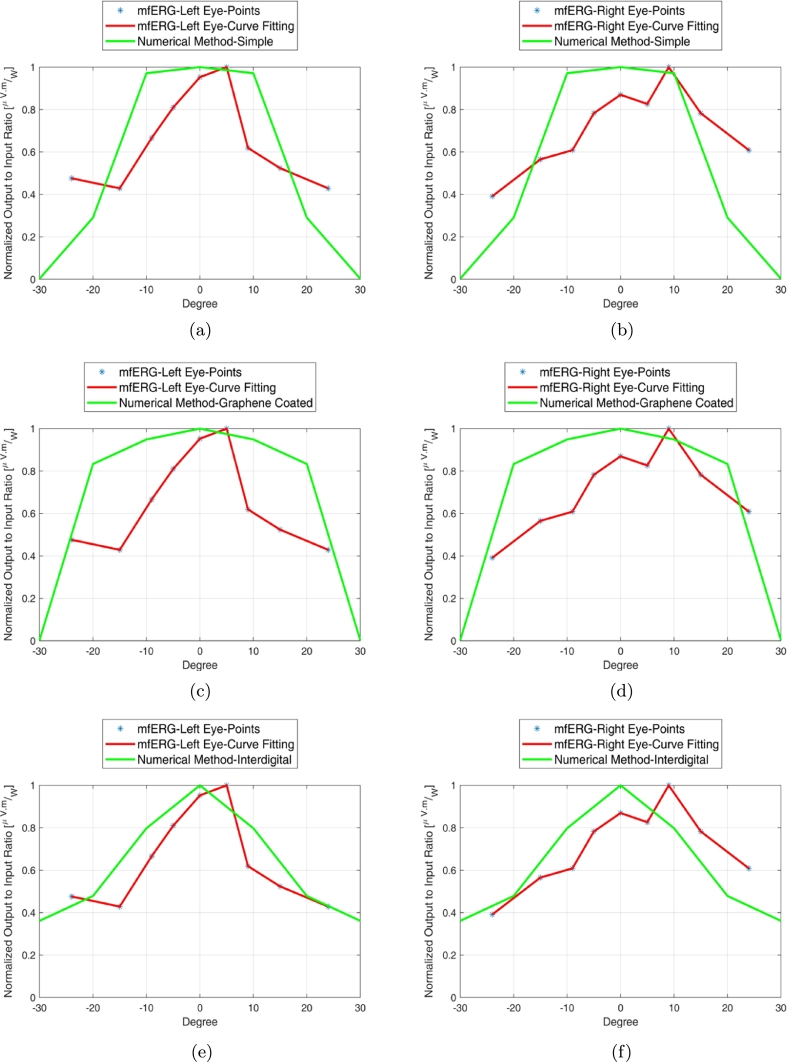
Comparison of numerical method array (at one frequency sample) with mfERG clinical and experimental results in normalized output to input ratio [$\frac{\mu V.m}{W}$] (a) simple photoreceptor array model-left eye, (b) simple photoreceptor array model-right eye, (c) graphene photoreceptor array model-left eye, (d) graphene photoreceptor array model-right eye, (e) interdigital photoreceptor array model-left eye, and (f) interdigital photoreceptor array model-right eye.

Fig. 14(a) illustrates the number and distribution of cones and rods in ${\text{mm}}^{2}$ with respect to the distance within retina. The cones and rods connection to the retina nerves has been shown in Fig. 14(b). The microscopic demonstration of cones and rods with a line scale of 10 μm has been shown in Fig. 14(c). The form of Fig. 14 shows that the arrangement of photoreceptor array should be like Fig. 15. There is infinite array of cone photoreceptor at the center of retina's field of view (0^∘^ to 5^∘^). The distribution of cones decreases from 5^∘^ to 15^∘^ meanwhile the distribution of rods increases in this area. The rods distribution will be maximum around 20^∘^-27^∘^ and afterwards it begins to decrease upto 30^∘^ and 70^∘^. The maximum input power (intensity) of incidence into photoreceptors is 0.5 $\frac{\text{W}}{{\mu \text{m}}^{2}}$. It is different for any introduced structures in this paper. The ratio between output (electric field at maximum possibility of local magnitude i.e. central locations) and input (intensity) of all structures (models) have been attained and then these values have been normalized to their maximum in each model based on the presented local constellation in Fig. 15. These normalized values (based on numerical method) are being compared with mfERG clinical and experimental ones as illustrated in Fig. 16. The values for simple, graphene coated and interdigital models have been shown in Figs. 16 (a) to (f). The best array model conforms to the mfERG results is the interdigital photoreceptor array model. The reason for this can be concluded by the shape of photoreceptor model as it has been designed with a near structure to the real photoreceptor of human eye at outer segment. The capacitance of outer segment in this model interacts with its inductance property and consequently affects the mentioned ratio (output to input) effectively. The interdigital cone photoreceptor has near 150 interdigital segments at outer segment (OS) and its rod photoreceptor has 1500 ones. It is noteworthy that the interdigital segment thickness and the periodic step of both cone and rod are 10 nm and 25 nm, respectively.

**Figure 17:**
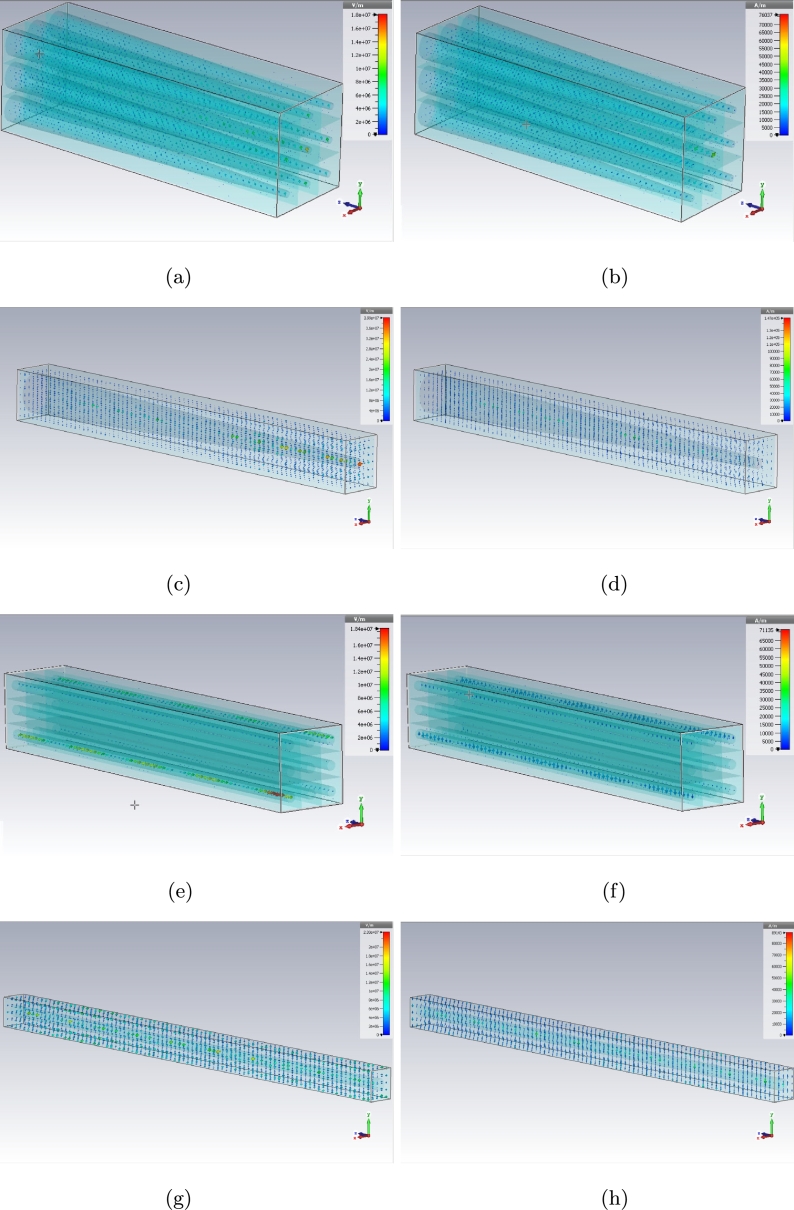
E (left) and H (right) plots for simple model (a), (b) cone - 3 × 3 array, (c), (d) cone - unit cell, (e), (f) rod - 3 × 3 array, and (g), (h) rod - unit cell.

**Figure 18:**
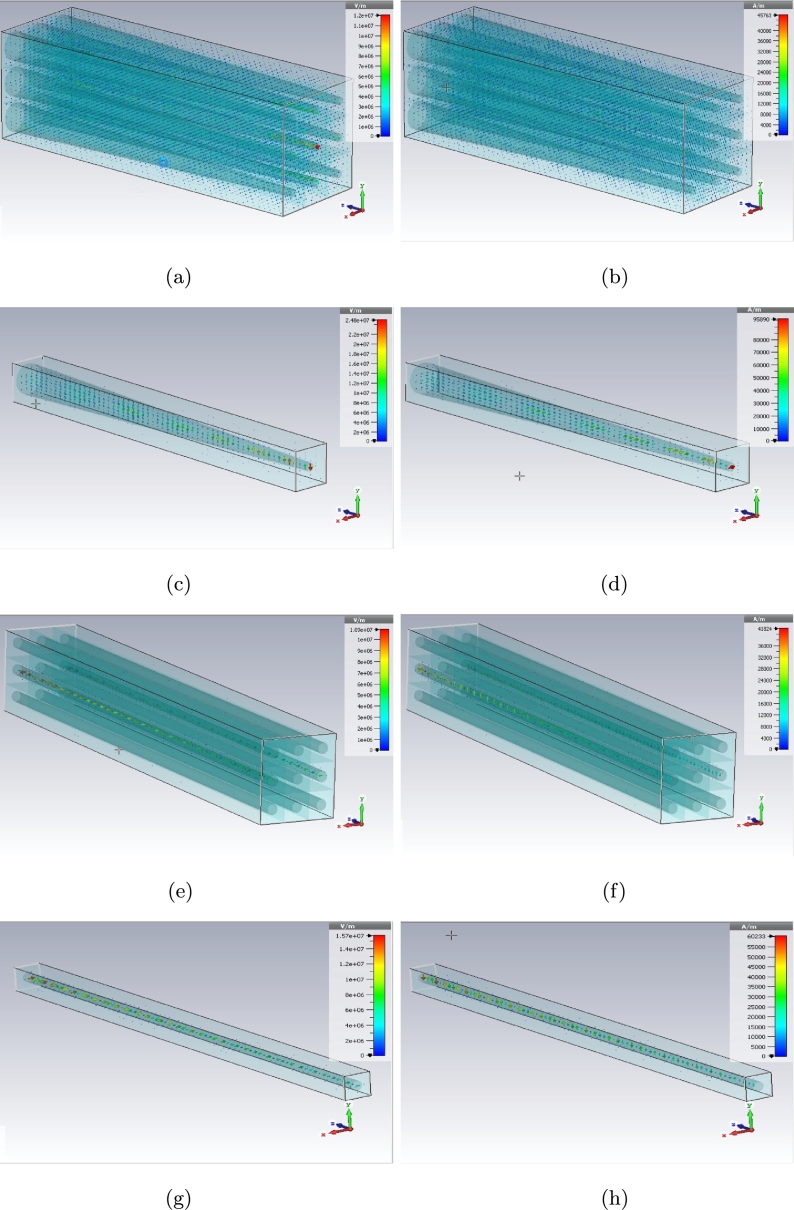
E (left) and H (right) plots for graphene coated model (a), (b) cone - 3 × 3 array, (c), (d) cone - unit cell, (e), (f) rod - 3 × 3 array, and (g), (h) rod - unit cell.

**Figure 19:**
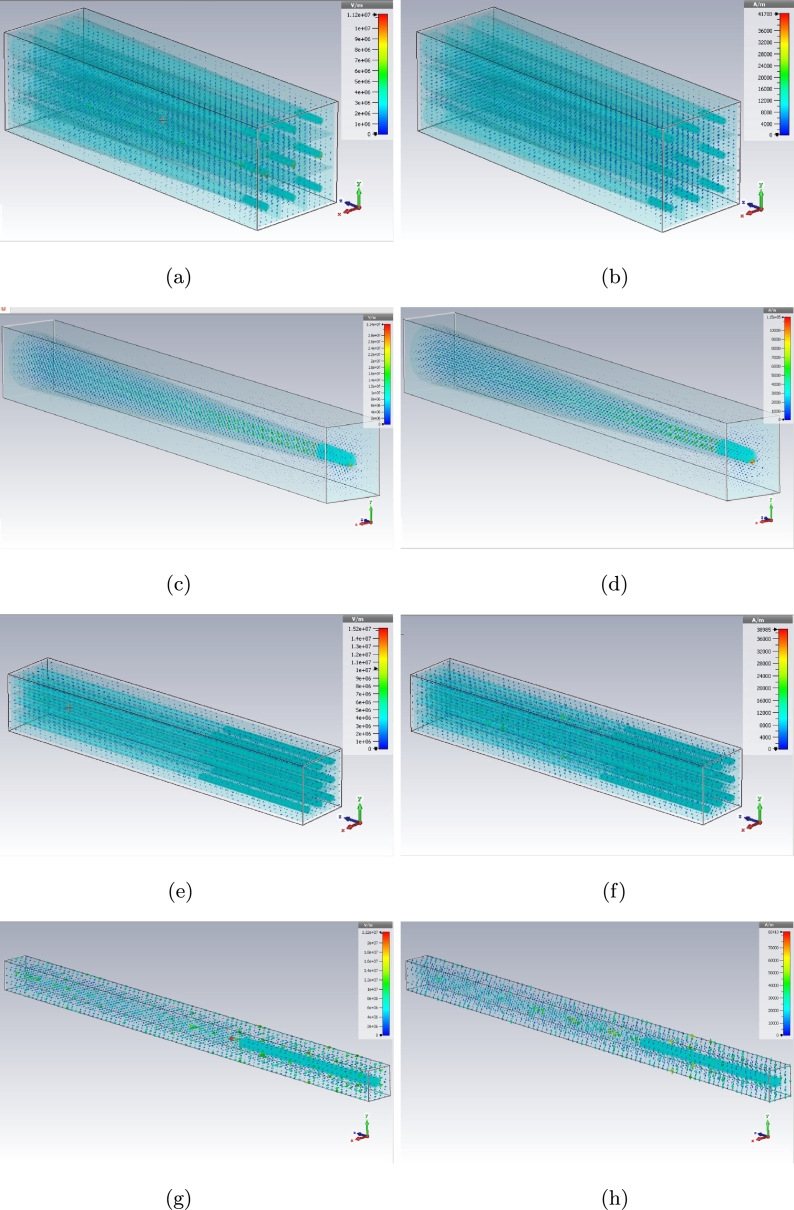
E (left) and H (right) plots for interdigital model (a), (b) cone - 3 × 3 array, (c), (d) cone - unit cell, (e), (f) rod - 3 × 3 array, and (g), (h) rod - unit cell.

The E and H fields of dielectric photoreceptors for every model can be presented here as these fields have been shown with maximum probability location for both conveying and receiving (on Z axis) the signals in the previous figures. Figure 17, Figure 18, Figure 19 are illustrating the electromagnetic fields on dielectric models for simple, graphene coated and interdigital ones, respectively.

There are E and H fields for simple cone and rod photoreceptors array in Fig. 17 (a) to (h). The fields for graphene coated photoreceptors array are in Fig. 18 (a) to (h) as well. The E and H fields of interdigital photoreceptors array model are being shown in Fig. 19 (a) to (h).

The impedance bandwidth parameter of DRA severely depends on its permittivity. Decreasing the dielectric constant of the model results in the decrement of the Q-factor and consequently increases the bandwidth of the resonant modes. Moreover, the resonant frequencies of low dielectric constant are higher than those of with the high permittivity one [18].

**Figure 20:**
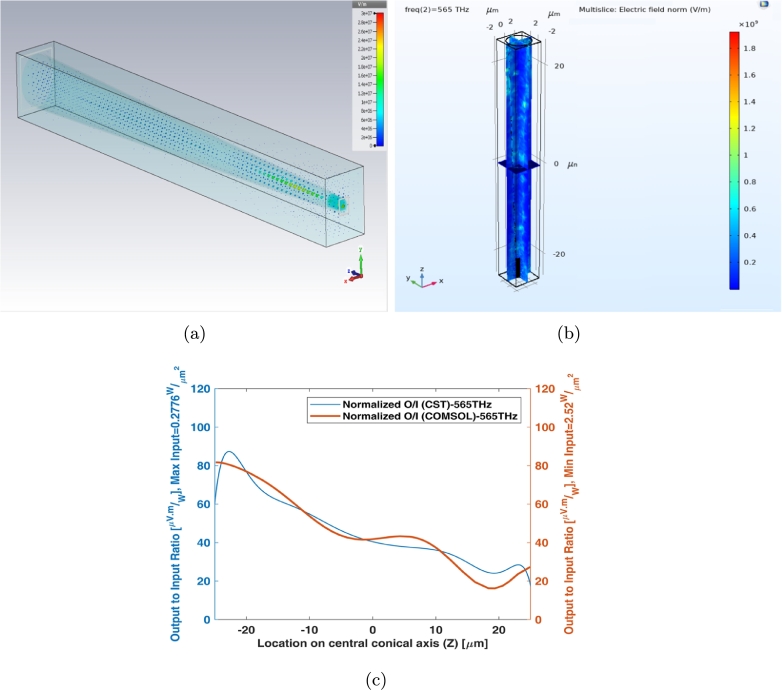
Single interdigital cone photoreceptor (a) E-field in CST MWS, (b) E-field in COMSOL multiphysics, and (c) output to input ratio (O/I) of numerical methods (near central fovea) on the central axis at specified sample frequency.

As one single cone photoreceptor is connected with one nerve through optic nerve, there is a comparison on Z axis for output to input ratio of interdigital cone model (the best suited model) by two numerical methods i.e. FIT (CST MWS) and FEM (COMSOL Multiphysics) at the determined sample frequency, 565 THz (Fig. 20). Fig. 20 (a) illustrates the electric fields of single interdigital cone photoreceptor in CST MWS whereas Fig. 20 (b) shows its form in COMSOL Multiphysics at one determined frequency. The maximum probability location for both conveying and receiving of the signals (on Z axis) are being shown for comparison with these two methods in Fig. 20 (c).

The aim of this research for showing these results is the inquiry of phototransduction process of photoreceptors mainly the received signals with respect to the input ones. So, dielectric transmission line can be more suitable for using in this note as well.

## 16 Conclusion

In this paper, electromagnetic dielectric resonator antenna (DRA) was presented for retina photoreceptors as a receiver antenna concept based on three models i.e., simple, graphene coated and interdigital structure at outer segment (OS) in order to model them at fovea with the respected angular spectrum. The proposed electromagnetic models based on dielectric resonator antenna (DRA) was analyzed for cones and rods of retina photoreceptors in human eye by utilizing the Finite Integral Method (FIM) associated with CST MWS. The results showed that these models were so fine for vision spectrum with a proper field enhancement in cone photoreceptors due to its sensitivity to light by the localized near field enhancement property. The results indicated fine properties of $S_{11}$ (return loss below -10 dB) with invaluable resonants in a wide range of frequencies from 405 THz to 790 THz (vision spectrum), adequate $S_{21}$ (insertion loss 3-dB bandwidth), very good field distribution for flowing the power. Finally, the array of each model was compared with clinical and experimental techniques, mfERG. It was shown that the results obtained from mfERG validate the numeric results. The best array model conformed by the mfERG results was the interdigital photoreceptor array model. The reason for this concluded by the shape of photoreceptor model as it was designed with a near shape to the real photoreceptor of human eye at outer segment. The interaction of capacitance and inductance properties of outer segment in this model was one of these effective impacts on the mentioned ratio.

## Ethical approval

The study followed the tenets of declaration of Helsinki and was approved by the Farabi eye hospital/Tehran University of Medical Science's Institutional Review Board (IRB) and written informed consents were obtained from patients (Ethics committee reference number: ERC/R/332).

## Funding

None.

## Informed consent

Informed consent was taken from all individual participants.

## CRediT authorship contribution statement

**Mahdi Norooz Oliaei:** Conceived and designed the experiments; Analyzed and interpreted the data; Contributed reagents, materials, analysis tools or data; Wrote the paper. **Hamid Riazi Esfahani:** Conceived and designed the experiments; Performed the experiments; Contributed reagents, materials, analysis tools or data; Wrote the paper. **MohammadSadegh Abrishamian:** Contributed reagents, materials, analysis tools or data; Wrote the paper.

## Declaration of Competing Interest

None declared.

## References

[1] Humayun M.S., Weiland J.D., Fujii G.Y., Greenberg R., Williamson R., Little J., Mech B., Cimmarusti V., Van Boemel G., Dagnelie G., de Juan E. (November 2003). Visual perception in a blind subject with a chronic microelectronic retinal prosthesis. Vis. Res..

[2] Caspi A., Dorn J.D., Mc Clure K.H., Humayun M.S., Greenberg R.J., McMahon M.J. (2009). Feasibility study of a retinal prosthesis: spatial vision with a 16-electrode implant. Arch. Ophthalmol..

[3] Humayun M.S., Dorn J.D., da Cruz Lyndon, Dagnelie G., Sahel J.A., Stanga P.E., Cideciyan A.V., Duncan J.L., Eliott D., Filley E., Ho A.C., Santos A., Safran A.B., Arditi A., Del Priore L.V., Greenberg R.J. (April 2012). Interim results from the international trial of second sight's visual prosthesis. Ophthalmology.

[4] Watterson William J., Montgomery Rick D., Taylor Richard P. (April 2018). Modeling the improved visual acuity using photodiode based retinal implants featuring fractal electrodes. Front. Neurosci..

[5] Watterson W.J., Montgomery R.D., Taylor R.P. (July 2017). Fractal electrodes as a generic interface for stimulating neurons. Sci. Rep..

[6] Watterson W.J. (2017).

[7] https://blogs.uoregon.edu/richardtaylor/files/2018/01/Fracret-Market-Analysis-xsdk30.ppsx.

[8] NoroozOliaei M., Riazi Esfahani H., Abrishamian M.S. (2022). Graphene coated dielectric resonator antenna for modeling the photoreceptors at visible spectrum. Heliyon (Cell Press).

[9] R. Milo, R. Philips, Cell Biology by the Numbers, ISBN 978-0-8153-4537-4, Taylor & Francis Group.

[10] Hood D.C., Odel J.G., Chen C.S., Winn B.J. (2003). The multifocal electroretinogram. J. Neuro-Ophthalmol..

[11] Hood D.C., Bach M., Brigell M., Keating D., Kondo M., Lyons J.S., Marmor M.F., McCulloch D.L., Palmowski-Wolfe A.M. (2012). International society for clinical electrophysiology of vision, ISCEV standard for clinical multifocal electroretinography (mfERG) (2011 edition). Doc. Ophthalmol..

[12] Khojasteh Hassan, Riazi-Esfahani Hamid, Khalili Pour Elias, Faghihi Hooshang, Ghassemi Fariba, Bazvand Fatemeh, Mahmoudzadeh Raziyeh, Salabati Mirataollah, Mirghorbani Masoud, Riazi Esfahani Mohammad (2020). Multifocal electroretinogram in diabetic macular edema and its correlation with different optical coherence tomography features. Int. Ophthalmol..

[13] Mazahery Tehrani Neda, Riazi-Esfahani Hamid, Jafarzadehpur Ebrahim, Mirzajani Ali, Talebi Hossein, Amini Abdulrahim, Mazloumi Mehdi, Roohipoor Ramak, Riazi-Esfahani Mohammad (2015). Multifocal electroretinogram in diabetic macular edema; correlation with visual acuity and optical coherence tomography. J. Ophthalmic Vis. Res..

[14] Miguel-Jimenez Juan M., Ortega Sergio, Boquete Luciano, Rodríguez-Ascariz José M., Blanco Román (2011). Multifocal ERG wavelet packet decomposition applied to glaucoma diagnosis. Biomed. Eng. Online.

[15] Wright Tom, Cortese Filomeno, Nilsson Josefin, Westall Carol (2012). Analysis of multifocal electroretinograms from a population with type 1 diabetes using partial least squares reveals spatial and temporal distribution of changes to retinal function. Doc. Ophthalmol..

[16] https://web.stanford.edu%3Eclass%3Earchive%3Estanford.lecture.04.pdf.

[17] Introduction human vision light, color, eyes, etc.. https://people.cs.umass.edu%3ETeaching%3Eppt%3E691A_UNIT_Light_1.ppt.pdfs.

[18] Keyrouz S., Caratelli D. (2016). Dielectric resonator antennas: basic concepts, design guidelines, and recent developments at millimeter-wave frequencies. Int. J. Antennas Propag..

